# Development of a Chimeric Vaccine Against *Pseudomonas aeruginosa* Based on the Th17-Stimulating Epitopes of PcrV and AmpC

**DOI:** 10.3389/fimmu.2020.601601

**Published:** 2021-01-21

**Authors:** Ying Wang, Xin Cheng, Chuang Wan, Jinning Wei, Chen Gao, Yi Zhang, Hao Zeng, Liusheng Peng, Ping Luo, Dongshui Lu, Quanming Zou, Jiang Gu

**Affiliations:** National Engineering Research Center of Immunological Products, Department of Microbiology and Biochemical Pharmacy, College of Pharmacy, Third Military Medical University, Chongqing, China

**Keywords:** chimeric vaccine, *Pseudomonas aeruginosa*, pulmonary infection, Th17 responses, epitope analysis

## Abstract

Pulmonary infection caused by *Pseudomonas aeruginosa* (PA) has created an urgent need for an efficient vaccine, but the protection induced by current candidates is limited, partially because of the high variability of the PA genome. Antigens targeting pulmonary Th17 responses are able to provide antibody-independent and broad-spectrum protection; however, little information about Th17-stimulating antigens in PA is available. Herein, we identified two novel PA antigens that effectively induce Th17-dependent protection, namely, PcrV (PA1706) and AmpC (PA4110). Compared to intramuscular immunization, intranasal immunization enhanced the protection of rePcrV due to activation of a Th17 response. The Th17-stimulating epitopes of PcrV and AmpC were identified, and the recombinant protein PVAC was designed and generated by combining these Th17-stimulating epitopes. PVAC was successfully produced in soluble form and elicited broad protective immunity against PA. Our results provide an alternative strategy for the development of Th17-based vaccines against PA and other pathogens.

## Introduction

Respiratory infections caused by *Pseudomonas aeruginosa* (PA) are a major health problem globally. Recently, with the emergence of multidrug resistant (MDR) PA strains, the clinical treatment of PA pneumonia has faced enormous challenges, especially for patients with cystic fibrosis (CF), non-CF bronchiectasis (nCFB), or chronic obstructive pulmonary disease (COPD), patients who are undergoing mechanical ventilation and patients with other pulmonary disorders (1, 2). To date, most PA vaccine candidates have been developed from LPS O antigens (3), polysaccharide-protein conjugates, outer membrane proteins (4) and the type III secretion system component PcrV (5). Despite substantial research efforts over the past fifty years, a vaccine licensed for clinical use has not yet been approved, and several challenges remain to be addressed.

One urgent problem to be solved is that the protective effects of most PA vaccines rely mainly on antibody-mediated opsonophagocytic killing and/or inhibition of toxicity. Phase II clinical trials of the PA vaccine IC43, a fused protein (OprF/I)-based vaccine, showed that the vaccine caused a significant increase in antibody titers in volunteers. However, the infection rate was not significantly decreased (4). Furthermore, although LPS O antigen-based vaccines can mediate high levels of immunity to PA, the protection is limited to strains that have specific LPS serogroups (3). It is increasingly clear that the activation of strong opsonophagocytic antibodies alone is insufficient for a successful vaccine against PA.

The contribution of Th17 immunity to the prevention of infection by pulmonary pathogens has gradually been recognized (6). Pulmonary Th17 cells participate in the recruitment of neutrophils, the release of antimicrobial peptides, IL-17–driven Th1 immunity and so on (7). These effectors provide immunity against a wide range of pathogens through the antibody-independent pathway (7). Therefore, inducing protective Th17 responses is of great importance when designing a vaccine against a pulmonary pathogen. Vaccine candidates that target Th17 responses have been identified and tested to protect against *Yersinia pestis* (8), *Mycobacterium tuberculosis* (9), *Bordetella pertussis* (10), *Streptococcus pneumoniae* (11), *Candida albicans* (12), and *Staphylococcus aureus* (13).

The Th17 response is also protective against pulmonary PA infection (14). To date, the number of Th17 cells and the concentration of IL-17A in the lungs have been shown to increase significantly as soon as 4 h after PA infection. Blocking the Th17 response leads to more severe pathological damage in the lung (14). A live attenuated PA vaccine was able to elicit serotype-independent protection in the mouse pneumonia model, and this protection relied not on specific antibodies but on Th17 responses (15). In an *in vitro* transcription system, a recombinant PcrH-PopB protein was found to stimulate the Th17 response and confer protection in mice (16, 17). In a previous study, we generated an optimized Th17-stimulating antigen, OprL, and investigated its serotype-independent protection (18). However, current Th17-stimulating vaccine candidates are difficult to apply in industrial settings because of potential safety risks, limited protection, and complicated production processes.

In this study, we produced ten soluble PA antigens in *E. coli* and identified two of them, rePcrV and reAmpC, that were able to induce effective protective Th17-dependent protection. Then, we screened the Th17-stimulating epitopes of rePcrV and reAmpC and generated a novel recombinant protein, PVAC, by rationally fusing the Th17-stimulating epitopes. Epitope-based PVAC-elicited immunity and protection were measured in mice.

## Materials and Methods

### Mice and Strains

Specific pathogen-free female C57BL/6 mice (six- to eight-week-old) were purchased from Beijing HFK Bioscience Limited Company (Beijing, People’s Republic of China). IL-17A gene knockout (IL-17A KO) mice (on a C57BL/6 background) were kindly provided by Richard A. Flavell (Yale University School of Medicine, New Haven, CT, USA). The mice were maintained under barrier conditions in a biohazard animal room. PA XN-1 (CCTCC M2015730) was isolated in Southwest Hospital in Chongqing, China, and deposited at the CCTCC (China Center for Type Culture Collection); its serotype is I. PA 464 and PA 451 were also isolated from Southwest Hospital. Their serotypes are F and A, respectively. PA ZNJ004 was isolated from the No.422 Hospital of the Chinese People’s Liberation Army, and its serotype is J.

### Production of Candidate Antigens in *E. coli*

To identify a possible soluble fragment of the target protein, its conserved domains were analyzed using the BLAST search tool (http://blast.ncbi.nlm.nih.gov/Blast.cgi). The nucleotide sequence codon-optimized for expression in *E. coli* was synthesized and cloned into pGEX-6p-1 (GE Healthcare) using the BamH I and Xho I restriction sites for expression in *E. coli* as a GST-fusion protein. The sequence of the recombinant plasmid was confirmed by DNA sequencing. Information on the candidate antigens is shown in **Table S1**. Then, protein expression in *E. coli* BL21 was induced by adding 0.3 mM IPTG to 1 L of cell culture in LB medium when the OD600 reached ~0.5. The cell culture was then incubated overnight with shaking at 150 rpm at 16°C. The cells were collected by centrifugation at 5,000 rpm for 20 min at 4°C and lysed by sonication in lysis buffer (20 mM phosphate buffer, pH 7.0, 150 mM NaCl) on ice. Then, the cell lysate was centrifuged at 12,000 rpm for 30 min, and the supernatant was collected and passed through a column of glutathione resin (NEB). After extensive washing with five-fold column volumes of washing buffer (20 mM phosphate buffer, pH 7.0, 1 M NaCl), PreScission Protease (GE Healthcare) was added and incubated overnight at 4°C. The recombinant proteins were then eluted from the glutathione resin, and protein impurities were further removed based on the different properties of the target proteins. The eluted proteins were finally concentrated to 4 to 6 mg/ml in buffer (PBS, pH 7.2) and identified by SDS-PAGE. The Keyhole limpet hemocyanin (KLH) was purchased from Genscript Company in Nanjing, China. The Endotoxin Cap Bestarose 4FF resin (Bestchrom) was used to remove the endotoxin in KLH and recombinant proteins in this study. The concentration of endotoxin in each protein was less than 10 EU/ml when determined by Limulus Amebocyte Lysate (LAL) quantitation kit (Pierce). Finally, these proteins were stored at −80°C for future assays.

### Immunization of Mice and Murine Pneumonia Model

In short, to evaluate the immune response, mice were divided equally into different groups and anesthetized with 0.12 ml of pentobarbital sodium (10 mg/ml) in 0.9% saline injected intraperitoneally (i.p.), then immunized intranasally with 20 μl of curdlan (10 mg/ml) and the corresponding antigen(25 μg/mouse) plus curdlan (10 mg/ml). The mouse immunization time points are 0, 14 and 21 days. Challenge or take tissue samples 14 days after the last immunization. Moreover, IL-17A knockout mice and wild-type (WT) mice were immunized to evaluate IL-17–associated protection. In another experiment, mice were injected i.p. with 2 mg of isotype control (RTK2071, Biolegend) or anti–IL-17 (TC11-18H10.1, Biolegend) antibody 2 days prior to challenge. For acute pneumonia challenge, the mice were anesthetized with pentobarbital sodium followed by the intratracheal injection of PA. The lethal doses of PA 464, PA XN-1, PA ZNJ004, and PA 451 were 2.5 × 10^7^, 1.0 × 10^7^, 3.5 × 10^6^, and 1.0 × 10^7^ CFU per mouse, respectively. The number of deaths caused by lethal infection was recorded every 12 h during a 7-day observation period post-challenge. In addition, the other mice were infected with a sublethal dose to investigate the protective mechanism. The sublethal doses of PA 464, PA XN-1, PA ZNJ004, and PA 451 were 3.5 × 10^6^, 1.3 × 10^6^, 5.8 × 10^5^, and 1.4 × 10^6^ CFU, respectively. The general information on these PA strains were included in **Table S2**.

### Isolation of Lung Mononuclear Cells and Intracellular Flow Cytometry Analysis

Lung tissues were chopped 14 days after the last immunization, and then digested with collagenase D (1 mg/ml, Gibco) and DNase I (10 mg/ml, Sigma-Aldrich) for 1 h at 37°C with agitation. Next, the lungs were passed through a 75 μm cell strainer to obtain a single-cell suspension. Then, the erythrocytes were removed by density gradient centrifugation with Percoll. Finally, lung mononuclear cells were obtained. After labeling with CFSE, the lymphocytes were incubated with the corresponding protein (0.5 μM) or Keyhole limpet hemocyanin (KLH), and stimulated with 5 U/ml IL-2 (PeproTech, Rocky Hill, NJ, USA) in complete RPMI-1640 medium. Half of the medium was removed when it turned yellow after incubation for about 2 days and then replaced with fresh medium. Then, the cells were stimulated with leukocyte activation cocktail with GolgiPlug for 4 h. The cell samples were washed and adjusted to a concentration of 5 × 10^5^ cells/ml with flow cytometry buffer (0.5% FBS in PBS). The samples were first stained for surface markers, followed by intracellular cytokine staining. For surface marker staining, the cells were incubated with the fluorochrome-conjugated monoclonal antibodies APC/Cyanine7 anti-mouse CD3 (17A2) and PerCP/Cyanine5.5 anti-mouse CD4 (GK1.5). For intracellular cytokine staining, the cells were processed with the Cytofix/Cytoperm Fixation/Permeabilization Kit (BD Biosciences) according to the manufacturer’s instructions. The samples were then incubated with fluorochrome-conjugated antibodies against APC anti-mouse IL-17A (TC11-18H10.1), PE anti-mouse IFN-γ (XMG1.2), and PE/Cy7 anti-mouse IL-4 (11B11). The above antibodies were all from Biolegend. Next, the samples were analyzed using BD FACSArray software™ on a BD FACSArray flow cytometer (BD Biosciences).

### ELISA

The levels of IgG were measured in serum samples collected 14 days after the last immunization. The 96-well ELISA plates were precoated with 100 μl of recombinant protein at 2 μg/ml overnight at 4°C. A total of 100 μl of serial two-fold dilutions of serum from each group of immunized mice was added to each well and incubated for 1 h at 37°C. The bound antibody was detected with goat anti-mouse IgG-HRP (Life Technology), and 100 μl of tetramethylbenzidine (Beijing ZSGB-BIO) substrate was added to each well to develop the color. The optical density at 450 nm (OD450) was measured. A well was considered positive when its OD450 was at least 2.1-fold higher than that of the negative control.

### Opsonophagocytic Killing Assay

First, HL-60 cells (ATCC, CCL-240) were differentiated into granulocyte-like cells in medium containing 100 mM N’N-dimethyl formamide for 5 days. Serum samples collected 14 days after the last immunization from immunized mice, and then were heat-inactivated (56°C, 30 min) and serially diluted with opsonization buffer (a mixture of 80 ml of sterile water, 10 ml of 10× Hank’s balanced salt solution, 10 ml of 1% gelatin, and 5.3 ml of fetal bovine serum). In 96-well plates, each well received 40 μl containing 4 × 10^5^ HL-60 cells, 10^3^ CFUs of XN-1 in 10 μl of opsonophagocytic buffer, 20 μl of serum, and 10 μl of 1% infant rabbit serum as a complement source. After incubation for 2 h, 10 μl of each sample was plated onto agar medium. The opsonophagocytic killing effect was defined as the reduction in CFUs after overnight incubation.

### Evaluation of Bacterial Load and Inflammation

The lungs of mice 24 h after challenge with a sublethal dose were collected, weighed, and homogenized in 1 ml of sterilized PBS. The homogenates were then plated on PIA plates and cultured at 37°C overnight. Then, the numbers of CFUs per gram of tissue were calculated from each plate. In addition, the concentrations of proinflammatory cytokines, such as IL-1β and TNF-α, in the supernatant were quantified by a Mouse Cytokine Quantification ELISA Test Kit (1210122, 1217202, Dakewei). The protocol was performed following the manufacturer’s (Dakewei) instructions. For histologic examination, the lungs were collected and fixed in neutral 10% formalin, embedded in paraffin, sectioned and stained with hematoxylin and eosin. The sections were observed at 100-fold magnification *via* light microscopy. Each lung section received a pathology score of 0 to 10 (from normal to severe) by a single pathologist according to the hemorrhage, edema, hyperemia, alveolar structure and neutrophil infiltration.

### Adoptive Transfer Experiments

Lung mononuclear cells were pooled from 15 immunized mice or 15 naïve mice and prepared as described above. The CD4^+^ T cells were sorted by negative selection (STEMCELL Technologies). A total of 5 × 10^6^ CD4+ T cells were transferred *via* intravenous injection. The mice were then challenged with PA XN-1 24 h after T cell transfer. Twenty-four hours after infection, pulmonary bacterial colonization and inflammation were detected as described above.

### Enzyme-Linked Immunospot (ELISPOT) Assay

A mouse IL-17A ELISpot PLUS kit (3521-4HPW-2, MabTech) was applied to measure peptide-specific IL-17-producing cells. In brief, lung mononuclear cells from immunized mice were prepared as described previously. Single-cell suspensions were added to the plate at a starting concentration of 1.5 × 10^6^ cells/well. The cells were cultured with 10 μg/ml peptide for 48 h and then tested according to the manufacturer’s instructions. The fold increase in the number of spot-forming cells (SFCs) was calculated with the following formula: Fold increase = (N_immunuzed_ − N_unimmunized_)/N_unimmunized_. N_immunuzed_ indicates the number of SFCs among lymphocytes from immunized mice, while N_unimmunized_ indicates the number of SFCs among lymphocytes from unimmunized mice.

### Statistical Analysis

The data are presented as the mean ± SE. The significance of differences was determined by an unpaired parametric test (Student’s t-test for two groups or one-way ANOVA for more than three groups). Bacterial burden was analyzed by the nonparametric Mann-Whitney test. The survival rate was analyzed by Kaplan-Meier survival curves. For pairwise comparisons among 3 or more groups, p values were adjusted by using Tukey’s test. SPSS15.0 and GraphPad Prism 8.0 were used for data analysis. Significance was accepted when P < 0.05.

## Results

### rePcrV and reAmpC Were Able to Induce a Th17 Response and Confer Protection in Mice

**Figure 1:**
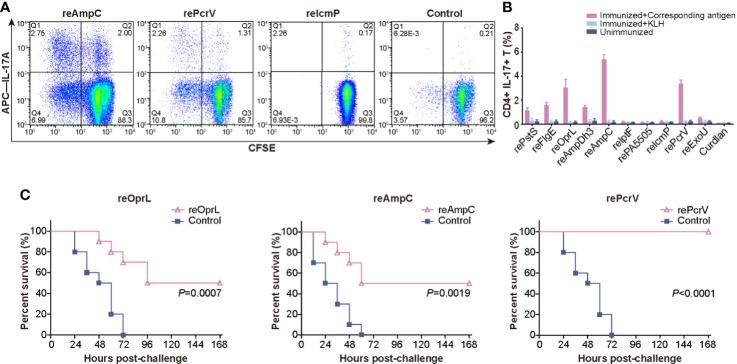
Screening for protective antigens that can induce a Th17 response. **(A)** Mice (n = 5) were intranasally immunized with the ten purified recombinant proteins, and lung lymphocytes were isolated and costimulated with the corresponding antigens. The representative dot plots show the frequency of CD4^+^ IL-17A^+^ T cells among lung lymphocytes from mice immunized with reAmpC, rePcrV, reIcmP, and the adjuvant curdlan. **(B)** The bar represents the percentage of CD4^+^ IL-17A^+^ T cells among lung lymphocytes from mice (n = 5) of the ten groups. The top three proteins were reAmpC (5.4%), rePcrV (3.4%) and reOprL (3.1%). **(C)** The survival of mice (n = 10) immunized with reOprL, reAmpC or rePcrV after challenge with a lethal dose of PA XN-1. All the immunized groups showed significantly better survival than did the control.

After critical review of the reported protective antigens and especially the data from the proteomic analysis of outer membrane vesicles (19), we tried to produce these 37 proteins in *E.coli* (**Table S1**). However only 10 of them were soluble and stable in PBS buffer, and were applied for further analysis (**Figure S1**). Mice were intranasally immunized with the ten purified recombinant proteins, and lung lymphocytes were isolated and costimulated with the corresponding antigens or KLH. Then, the percentage of CD4^+^ IL-17A^+^ T cells was measured to identify the antigens that efficiently induced a Th17 response. Immunization with reOprL was able to induce a Th17 response, which was consistent with previous observations (**Figure 1A**) (18). In addition, immunization with two recombinant proteins, namely, reAmpC and rePcrV, was capable of eliciting a Th17 response. The percentage of CD4^+^ IL-17A^+^ T cells in the reAmpC and rePcrV groups was significantly higher than that in the reOprL group (**Figure 1B**). KLH stimulation did not significantly increase the percentage of CD4^+^ IL-17A^+^ T cells. Next, we tested the protection induced by the ten candidates *via* intratracheal challenge with a lethal dose of PA XN-1. As shown in **Figure 1C**, the survival rate of the reOprL group was 50%, which was significantly higher than that of the curdlan (*P* = 0.0007) group. The survival rate of the reAmpC-immunized mice was also significantly higher than that of the control (P = 0.0019). No mice in the adjuvant control group survived more than 60 h. Surprisingly, the survival rate of the rePcrV vaccination group was as high as 100%, which was also significantly higher than that of the control (*P*<0.0001) group. The above results indicate that intranasal vaccination with reAmpC or rePcrV not only induces a robust Th17 response but also provides protective effects.

### Compared With Intramuscular Vaccination, Intranasal Vaccination With rePcrV Enhanced Protection

**Figure 2:**
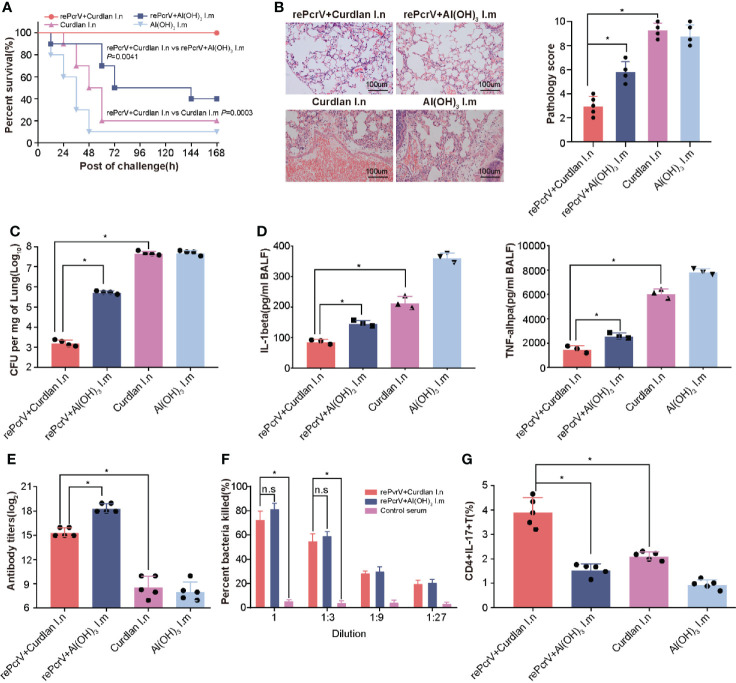
Compared with intramuscular vaccination, intranasal vaccination with rePcrV enhanced protection. **(A)** Mice were immunized with rePcrV intranasally (i.n.) or intramuscularly (i.m.). The bar indicates the survival of mice (n = 10) after intratracheal injection with a lethal dose of PA XN-1. The survival rate of the i.n. group was significantly higher than that of the i.m. group. **(B)** HE staining of lungs from immunized mice 24 h after infection with a sublethal dose of PA XN-1. Images were captured at 100× magnification. **(C)** Evaluation of the bacterial load in the lung in PA XN-1−infected mice (n = 4). The bar represents the average and SE of the log CFU of PA XN-1 per mg of lung tissue. **(D)** Quantitative measurement of TNF-α and IL-1β in the lungs (n = 3). The data are shown as the mean ± SE. **(E)** The bar represents the titer of anti-rePcrV IgG antibodies in the serum of immunized mice (n = 5). **(G)** The opsonophagocytic killing activity of the serum from immunized mice. Serum samples from immunized mice were diluted and incubated with PA XN-1. The bar represents the percentage of killed bacteria in a series of dilutions. The data are presented as the mean ± SE. **(F)** Frequency of CD4^+^ CD17^+^ T cells in the lungs of mice two weeks after the last immunization. The bar represents the frequency of the cells in each group. The “*” indicates a significant difference at *P* < 0.05. The “n.s” means “no significant difference”.

Intramuscular (i.m.) immunization with the PcrV protein formulated with Al(OH)_3_ has been studied extensively (5, 20), we determined whether intranasal (i.n.) immunization could improve the efficiency of immunization. As expected, the survival rate of the i.n. group was significantly higher (*P* = 0.0041) than that of the i.m. group (**Figure 2A**). Subsequently, the immunized mice were challenged with a sublethal dose of PA XN-1 to explore the protective mechanism. Compared with the rePcrV i.m. group and the curdlan i.n. control group, the rePcrV i.n. group showed significant reductions in pathology score, which was characterized according to the cell infiltration, hemorrhage, alveolar collapse and tissue damage (**Figure 2B**). Interesting, more infiltrate cells but less pathogenic lesions was observed rePcrV i.n. group. This observation indicates the participation of protective resident memory T cells, which was usually found in intranasal delivered vaccines (21).

In addition, the bacterial loads of the rePcrV i.n. group were significantly lower than those of the rePcrV i.m. (*P*<0.0001) and curdlan i.n. control groups (P = 0.0002) (**Figure 2C**). Furthermore, compared with the rePcrV i.m. and curdlan i.n. groups, the levels of the proinflammatory cytokines TNF-α and IL-1β in the BALF of the rePcrV i.n. group were significantly lower (**Figure 2D**). These results indicated that the improved protection of i.n. immunization with rePcrV might be due to the decrease in the bacterial load, the secretion of proinflammatory cytokines and the increase in inflammation in the lung.

In addition, the immune response following rePcrV vaccination was determined. Surprisingly, the titer of anti-PcrV antibodies in the i.n. group was significantly lower (*P*<0.0001) than that in the i.m. group (**Figure 2E**). However, no significant difference in the opsonophagocytic activity of anti-PcrV antibodies was observed between the two immunization routes (**Figure 2F**). These findings led us to measure the pulmonary Th17 response induced by rePcrV. As shown in **Figure 2G**, the percentage of CD4^+^ IL-17A^+^ T cells in the lung of the rePcrV i.n. group was significantly higher (*P*<0.0001) than that of the rePcrV i.m. group. These results suggested that lung Th17 cells could be responsible for the enhanced protection induced *via* the intranasal route.

### The Enhanced Protection Induced by Intranasal Vaccination With rePcrV Depends on the Th17 Response

**Figure 3:**
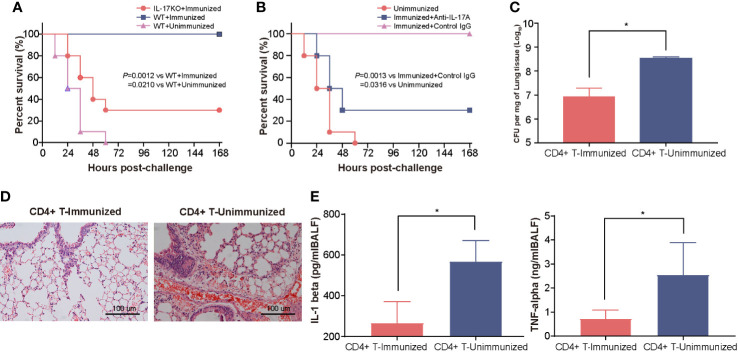
The enhanced protection of intranasal vaccination with rePcrV depends on the Th17 response. **(A)** WT (wild-type) mice or IL-17A knockout mice (n = 10) were immunized with rePcrV and then intratracheally challenged with a lethal dose of PA XN-1. The bar represents the survival of the mice in each group. **(B)** WT mice were immunized with rePcrV and administered anti-IL-17A antibodies (n = 10) and with isotype control IgG as a control. The bar represents the survival rate of the two groups at each observation point. **(C)** CD4+ T cells from the lungs of rePcrV-immunized mice were transferred into naïve WT mice. Then, the mice were challenged with a sublethal dose of PA XN-1. The bar represents the average and SE of the log CFU of PA XN-1 per mg of lung tissue (n = 4). **(D)** HE staining of the lungs of mice after infection with a sublethal dose of PA XN-1. Images were captured at 100× magnification. **(E)** Quantitative measurement of TNF-α and IL-1β in the BALF of mice (n = 3). The data are shown as the mean ± SE. The “*” indicates a significant difference at *P* < 0.05.

To clarify the contribution of the Th17 response to the increase in protection, we first tested the protection induced in IL-17A KO mice after intranasal vaccination with rePcrV. As few as 30% of immunized IL-17A KO mice survived, which was significantly lower (*P* = 0.0012) than the survival rate observed in WT (wild-type) mice (**Figure 3A**). Similar results were observed in immunized WT mice after treatment with anti−IL-17A mAbs. The survival was significantly decreased (*P* = 0.0013) in mice injected with IL-17A mAbs compared with mice injected with control mouse IgG (**Figure 3B**). Next, we isolated CD4^+^ T cells from the lungs of mice immunized intranasally with rePcrV and then adoptively transferred them into naïve mice. The mice were challenged with PA XN-1 to further evaluate CD4^+^ T cell-mediated protection. We found that bacterial colonization in the lungs of the mice given CD4^+^ T cells was significantly lower (*P* = 0.0025) than that of the mice in the control group (**Figure 3C**). Similarly, pathological observation showed that lesions such as pulmonary hemorrhage and inflammatory cell infiltration were significantly reduced after injection of CD4^+^ T cells (**Figure 3D**). Finally, we tested the concentration of proinflammatory factors (TNF-α and IL-1β) in the BALF. Similarly, adoptive transfer of CD4^+^ T cells from the immunized group significantly (*P* = 0.0390, *P* = 0.0060) reduced the concentration compared with that of the control group (**Figure 3E**). These results suggest that the Th17 response is indispensable for the enhanced protection induced by intranasal vaccination with rePcrV.

### The Protection Mediated by reAmpC Relies on the Th17 Response

**Figure 4:**
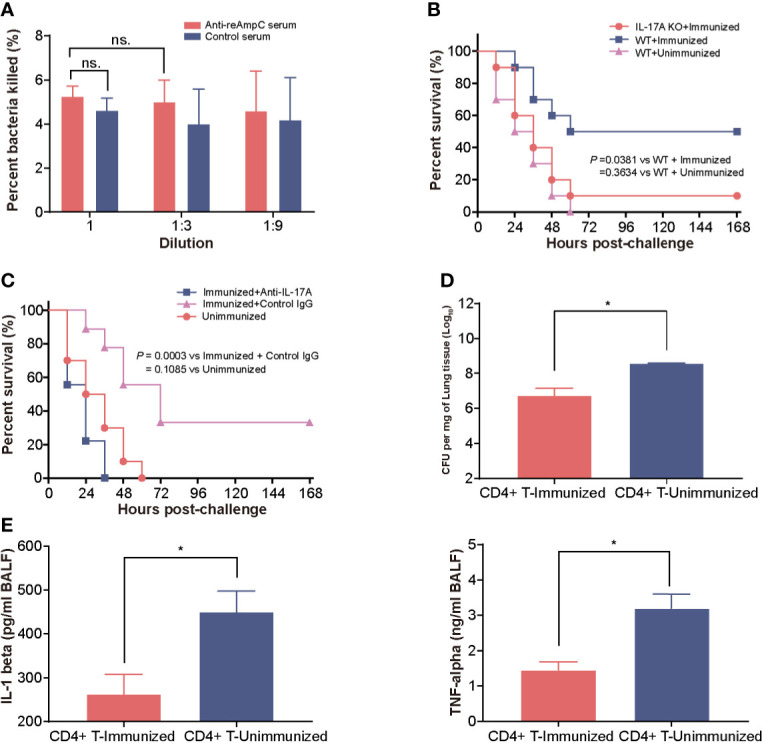
The protection mediated by reAmpC relies on the Th17 response. **(A)** The opsonophagocytic killing activity of serum from immunized mice. The bar represents the percentage of killed bacteria in a series of dilutions. The data are presented as the mean ± SE. **(B)** WT (wild-type) mice or IL-17A knockout mice were immunized with reAmpC (n = 10) and then intratracheally challenged with PA XN-1. The bar represents the survival of mice in each group. **(C)** WT mice or IL-17A knockout mice (n = 10) were immunized with reAmpC and then intratracheally challenged with a lethal dose of PA XN-1. The bar represents the survival of mice in each group (n = 10). **(D)** CD4+ T cells from the lungs of reAmpC-immunized mice were transferred into naïve WT mice. Then, the mice were challenged with a sublethal dose of PA XN-1. The bar represents the average and SE of the log CFU of PA XN-1 per mg of lung tissue (n = 4). **(E)** Quantitative measurement of TNF-α and IL-1β in the BALF of mice (n = 3). The data are shown as the mean ± SE. The “*” indicates a significant difference at *P* < 0.05. The “n.s” means “no significant difference”.

First, we measured the opsonophagocytic killing activity of anti-reAmpC antibodies. However, no significant difference in bacteria-killing rates was observed between sera from reAmpC-immunized mice and unimmunized mice, which suggest a limited contribution of reAmpC-specific antibodies to preventive effects (**Figure 4A**). Building on these findings, we next aimed to clarify whether Th17 immunity contributes to protection. As expected, the survival rate of immunized wild-type mice was 50%, while that of immunized IL-17A KO mice was only 10%. All WT mice that were not immunized died within 60 h after the challenge (**Figure 4B**). In another setting, the immunized mice were treated with anti-IL-17A mAbs to block the Th17 response before the bacterial challenge. The results showed that the survival decreased significantly (*P* = 0.0003) in mice injected with anti−IL-17A mAbs compared with control mice injected with nonspecific mouse IgG (**Figure 4C**). Additionally, the CD4^+^ T cells of reAmpC-immunized mice were isolated and adoptively transferred to naïve mice to evaluate their protective effects. The number of colonizing bacteria in the lungs of mice that received CD4^+^ T cells from the lungs of reAmpC-immunized mice was significantly (*P*<0.0001) lower than that in the lungs of unimmunized mice (**Figure 4D**). Moreover, a similar trend was observed for the levels of the proinflammatory factors IL-1β and TNF-α in the BALF (**Figure 4E**). Taken together, these findings showed that the protection induced by reAmpC relies on the pulmonary Th17 response.

### Mapping of Epitopes in rePcrV and reAmpC That Stimulate the Th17 Response

**Figure 5:**
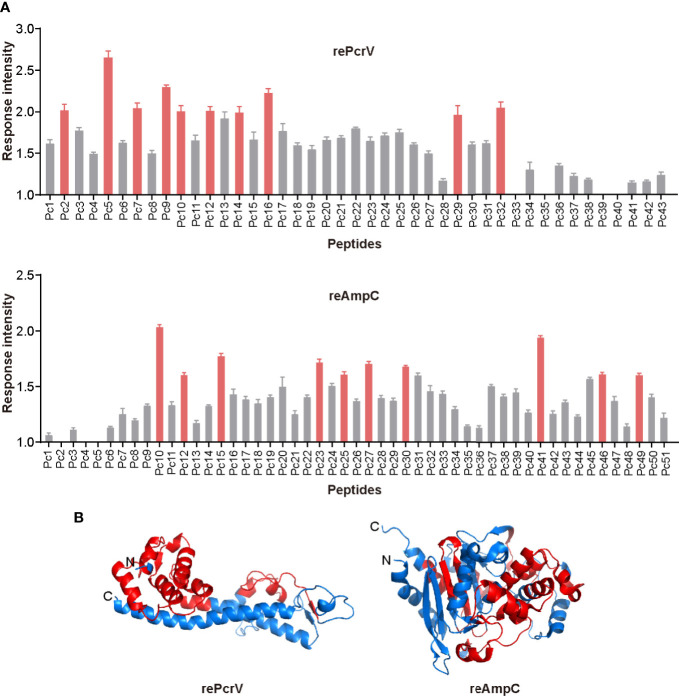
Mapping of epitopes in rePcrV and reAmpC that stimulate a Th17 response. **(A)** The lung lymphocytes of mice immunized with rePcrV (upper) and reAmpC (lower) were isolated and cocultured with the corresponding peptide library. The number of IL-17−secreting cells induced by each peptide was detected by ELISpot. The bars represent the mean and SE of the fold increase in SFCs (spot-forming cells). The top ten peptides are shown in red. **(B)** Cartoon image of the 3D structure of PcrV (left) and AmpC (right). The model of PcrV was built with the I-TASSER Suite, while the model of AmpC was obtained from the Protein Data Bank (PDB: 4GZB). The images were generated with PyMol 2.4 software. The regions corresponding to the top ten peptides are shown in red. The Th17-stimulating epitopes in rePcrV accumulated at its N-terminus, while those of reAmpC spanned the whole protein.

To screen for epitopes that can stimulate Th17 responses, we isolated lung lymphocytes from immunized mice and cultured them with synthesized peptides from the library of rePcrV and reAmpC (**Tables S3** and **S4**). IL-17−secreting cells were then identified by ELISPOT assay. The distribution of Th17-stimulating epitopes in rePcrV and reAmpC is shown in **Figure 5A**. The highest fold change in the number of spot-forming cells (SFCs) was clearly observed for Pc5 (LSEAQVLKALAWLLAANP), which had approximately 2.5-fold more SFCs than the unimmunized mice. Furthermore, most of the Th17 stimulation epitopes are located at the N-terminus of rePcrV (**Figure 5B**). In addition, the top 10 most potent epitopes of reAmpC for promoting the secretion of IL-17A are shown in red (**Figure 5A**, lower panel). P10 (FTATLAGYALTQDKMRLD) and P41 (AGNSTPMALQPHRIARLP) were the most predominant epitopes in reAmpC, with approximately 2.0- and 1.9-fold more SFCs than the unimmunized mice, respectively. Unlike rePcrV, the epitopes in reAmpC did not accumulate in one region but spanned the whole molecule (**Figure 5B**).

### Vaccination With PVAC Protects Against Four PA Strains of Different Serotypes

**Figure 6:**
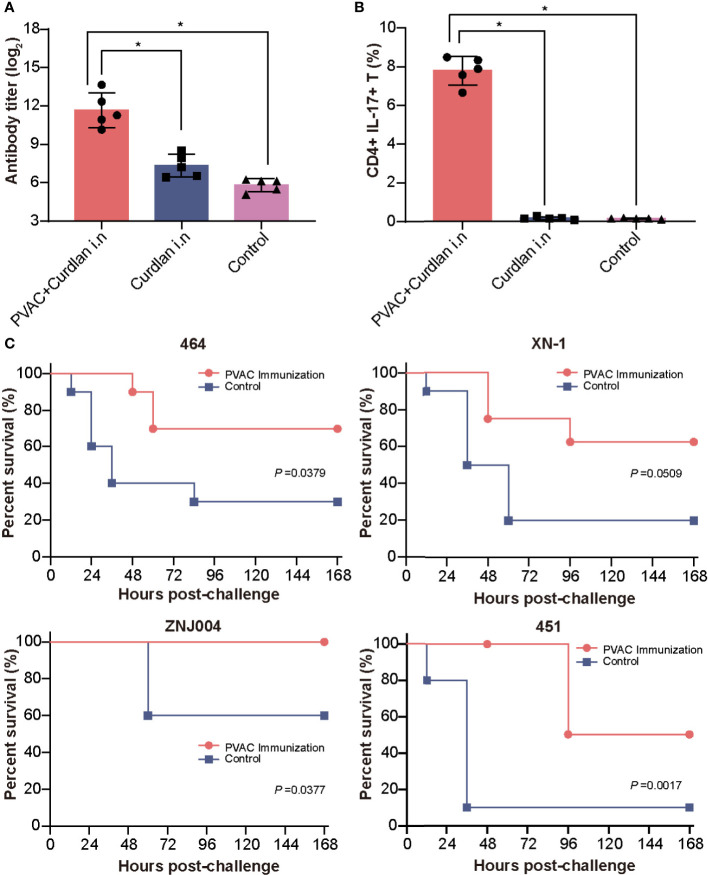
Vaccination with PVAC protects against PA strains of four different serotypes. **(A)** PVAC, which is composed of the N-terminus of rePcrV (27Glu-126Gly) connected to full-length reAmpC, was produced and used to immunize mice. The bar represents the titer of anti-PVAC IgG in the serum of immunized mice (n = 5) **(B)** The bar represents the frequency of CD4^+^ CD17^+^ T cells in the lungs of immunized mice. **(C)** PVAC-immunized mice were challenged *via* intratracheal injection of PA-464, PA XN-18 PA ZNJ004, and PA-451. The survival rates of the four immunized groups were significantly higher than that of the control group. The “*” indicates a significant difference at *P* < 0.05.

Building on these findings, we hypothesized that a combination of Th17-stimulating epitopes could induce more robust protection. After several attempts, we successfully produced the soluble recombinant protein PVAC, which was composed of the N-terminus of rePcrV (27Glu-126Gly) connected to full-length reAmpC with the flexible linker GSGGSG (**Figure S3**). Intranasal immunization with PVAC could also induce a humoral immune response, as indicated by an elevated titer of PVAC-specific antibodies in the serum (**Figure 6A**). More importantly, the number of CD4^+^ IL17^+^ T cells in the lung increased dramatically after immunization, which suggested a potent Th17 response (**Figure 6B**). Next, to determine whether PVAC could provide broad protection, immunized mice were challenged with clinical PA isolates of different serotypes: PA 464, PA XN-1, PA ZNJ004, and PA 451. The serotypes of these four strains were F, I, J and A, respectively. The survival rate of immunized mice was 70%, 60%, 100%, and 50% after challenge with PA 464, PA XN-1, PA ZNJ004, and PA 451, respectively (**Figure 6C**). The survival rates of the four immunized groups were significantly higher than that of the control group, which indicates that broad protection was induced by PVAC. Collectively, these results show that the fusion protein PVAC was able to effectively induce a humoral immune response and a Th17 response, which proved that broad protection was induced in the mouse pneumonia model.

## Discussion

One major challenge for developing a PA vaccine is the hypervariation of its genome, which requires a “universal vaccine” to ensure broad protection (22). By targeting the Th17 response, serotype-independent protection was elicited by attenuated PA (15), recombinant reOprL (18) or PopB coexpressed with PcrH (16). Despite these efforts, a Th17-based universal PA vaccine has yet to be attained. In fact, PopB-PcrH is too difficult to produce, and the protection induced by reOprL is limited. In this study, we generated recombinant PVAC according to the Th17-stimulating epitopes of PcrV and AmpC. The results showed that recombinant PVAC was efficiently produced in *E. coli* and was able to induce broad protective immunity against PA. Our results provide additional data for a Th17-based vaccine and will benefit the future development of PA vaccines.

One interesting finding is that the serum anti-PcrV antibodies induced by intranasal immunization were opsonophagocytic and protective, which is very different from the antibodies induced by other Th17-stimulating antigens, such as reOprL, reAmpC and PopB (17, 18). One possible explanation is that PcrV is the critical component of the type three secretion system, which is located in the outer membrane of bacteria (23). Thus, the antibodies may be able to bind to PcrV and block the effects of toxins secreted by the secretion apparatus. In addition, anti-PcrV antibodies promote the internalization of PA and the localization of ingested PA into acidified vacuoles, which ultimately stimulates antibody-dependent cellular cytotoxicity (20, 24). To date, two therapeutic antibodies targeting PcrV, KB001 and MEDI3902 have been proven to be protective in animal models and are being tested in clinical trials (24, 25). As a result, it is not surprising that elevated protection is mediated by intranasal immunization with PcrV because Th17 cells and antibodies induce simultaneous effects.

In this study we found that the PVAC, which contains the predominant Th17-stimulating epitopes, could provide wide-spectrum protection against PA. However, the protective efficacy of PVAC was lower than that of rePcrV in the case of PA XN-1. The decrease of efficacy is possibly due to the change of humoral response because PVAC contains the most Th17-stimulating epitopes of PcrV. Since PcrV is a key component of the T3SS, one mechanism for anti-PcrV immunity is based on the inhibition of the cytotoxic effects of T3SS toxins (5, 26). The epitopes recognized by the protective antibodies are predominantly located in the 144–257 region (27, 28). However, these amino acids are not present in the PVAC. Consequently, antibodies against this region are missing in mice immunized with PVAC, resulting in decreased protection. These data suggest that protective epitopes recognized by B-cells should be integrated to improve the protective effect of the vaccine in future studies.

The variability of vaccine targets is closely correlated to vaccine efficacy. The *pcrV* gene was found to be conserved among diverse PA isolates (29). AmpC is a class C lactamase that provides resistance against antipseudomonal cephalosporins, and it is present in most PA strains (30). Epidemiological studies showed that mutations in AmpC commonly increase the resistance to antipseudomonal cephalosporins, which potentially impairs the efficacy of PVAC-mediated protection (31, 32). However, AmpC mutations are usually individual amino acid substitutions, which only affect a small minority of epitopes. In addition, these mutations were only detected in a small proportion (about 1%) of clinical PA isolates, which is different from the extensive diversity of PA habitats (31). As a result, we concluded that AmpC mutations are likely to have a very limited effect on the protection mediated by PVAC.

One bottleneck for the development of Th17-based vaccines is the lack of effective adjuvants. Traditional aluminum adjuvants preferentially induce Th2 and antibody responses, although it is possible for these adjuvants to promote a Th17 response by activating the inflammasome *via* the release of uric acid (33). Several cytokines, such as IL-1β, IL-6, and IL-23, were found to be able to induce or enhance a strong Th17 response (34, 35). Additionally, recombinant *E. coli* heat-labile enterotoxin and cholera toxin were able to activate dendritic cells and enhance the secretion of cytokines to induce a Th17 response (36, 37). Another type of Th17 adjuvant is TLR receptor agonists. For example, a TLR4 agonist, monophospholipid A-trehalose dimycolate (TDM), is capable of enhancing the differentiation of Th17 cells. In this study, the TLR4 agonist curdlan, which has previously been proven to be able to induce a Th17 response, was applied as an adjuvant. However, no adjuvant that preferentially generates a Th17 response is currently available due to potential toxicity or insolubility, which highlights an urgent need for novel adjuvants for the development of Th17-based vaccines.

One major concern related to Th17-based vaccines is safety. Despite their protective effects against pathogens at mucosal sites, Th17 responses have been proven to be pathogenic in many diseases (38). For example, Th17 cells can infiltrate tissues and secrete proinflammatory TNF-α and GM-CSF, which exacerbate the pathogenesis of rheumatoid arthritis (RA), multiple sclerosis (MS) and other autoimmune diseases (39, 40). In addition, long-lived Th17 cells may be the reason for the persistence of HIV infection (41, 42). Importantly, accumulating data indicates that Th17 cells are essential for the pathogenesis of chronic obstructive pulmonary disease (COPD) and cystic fibrosis (CF) (43). However, patients with COPD or CF are a major target population of the PA vaccine due to their susceptibility to this infection. Thus, careful assessment of the safety of the Th17-based PA vaccine is required before immunization of patients with COPD, CF and autoimmune diseases.

The immunogenicity of multi-epitope vaccines may be restricted when applied to humans because of the difference of major histocompatibility complex (MHC) between humans and mice. To bridge this gap, transgenic HLA mice were applied as reliable animal models in pre-clinical investigation of vaccines (44). For example, a vaccine against *Coccidioides posadasii* was generated using human Th17-driven epitopes (45), and its protective effect was evaluated in humanized HLA-DR4 transgenic mice (46). Adequate investments are needed to evaluate the efficacy of PVAC in HLA class I and class II humanized transgenic mice in order to develop a human vaccine against PA in the future.

## Data Availability Statement

The original contributions presented in the study are included in the article/**Supplementary Material**. Further inquiries can be directed to the corresponding authors.

## Ethics Statement

The animal study was reviewed and approved by the Animal Ethical and Experimental Committee of the Third Military Medical University.

## Author Contributions

YW, XC, CW, JW, and CG performed the experiments. YZ, LP, PL, and DL supervised the experiments. YW and JG wrote and revised the manuscript. HZ and QZ designed the project and supervised the experiments. All authors contributed to the article and approved the submitted version.

## Funding

This research was supported by the National Natural Science Foundation of China (81772155 and 81571621) and Natural Science Foundation of Chongqing (CSTC2020JCYJ-MSXM2301).

## Conflict of Interest

The authors declare that the research was conducted in the absence of any commercial or financial relationships that could be construed as a potential conflict of interest.
