# Supplementary figures and images for: Development of a Chimeric Vaccine Against Pseudomonas aeruginosa Based on the Th17-Stimulating Epitopes of PcrV and AmpC
Source: Front Immunol. 2021 Jan 21;11:601601. doi: 10.3389/fimmu.2020.601601 (PMC7859429; doi:10.3389/fimmu.2020.601601)

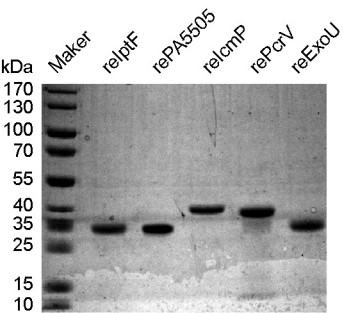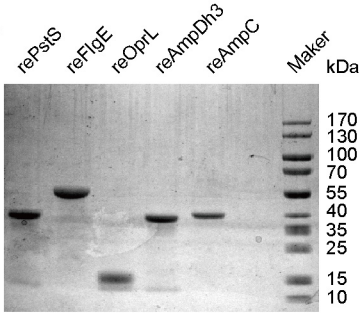

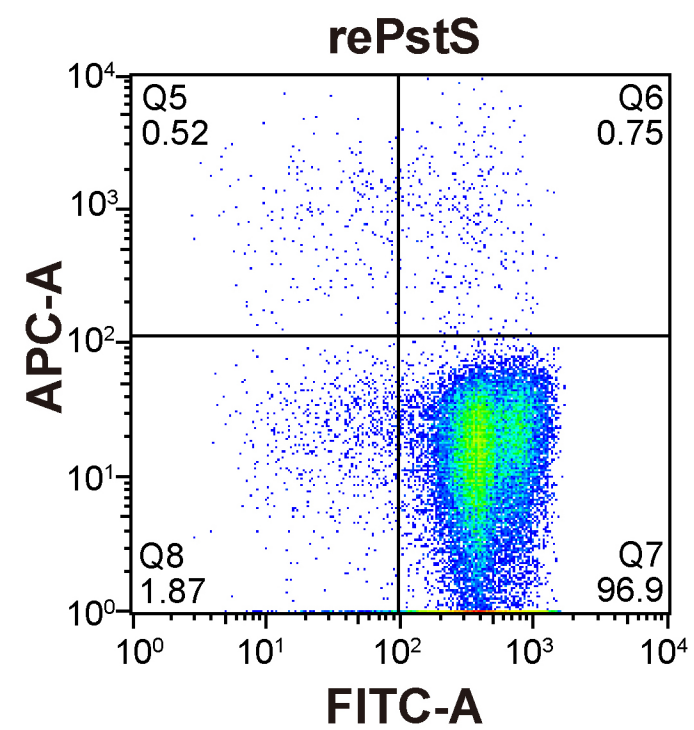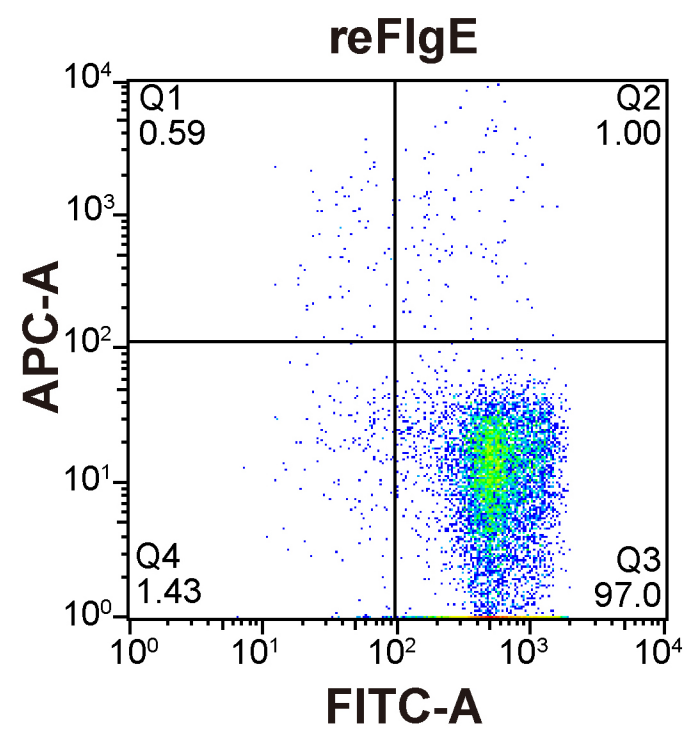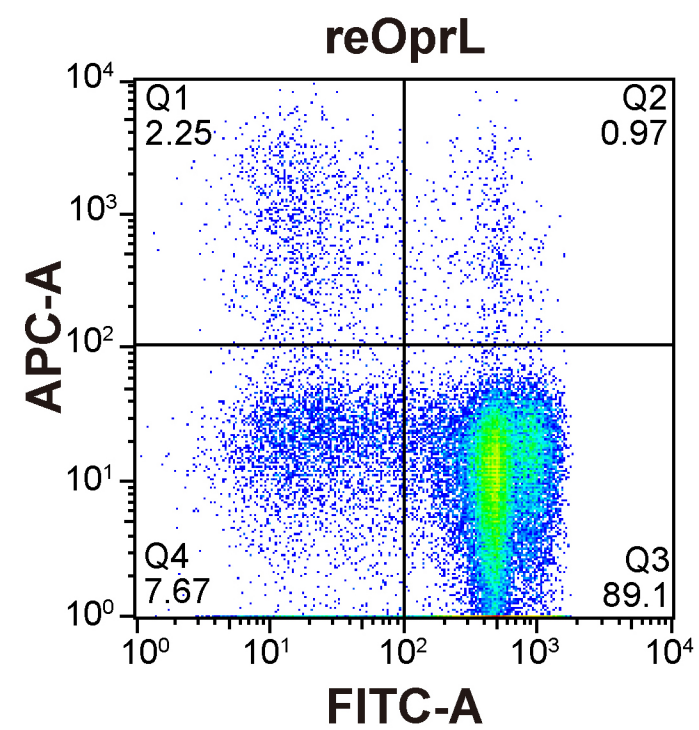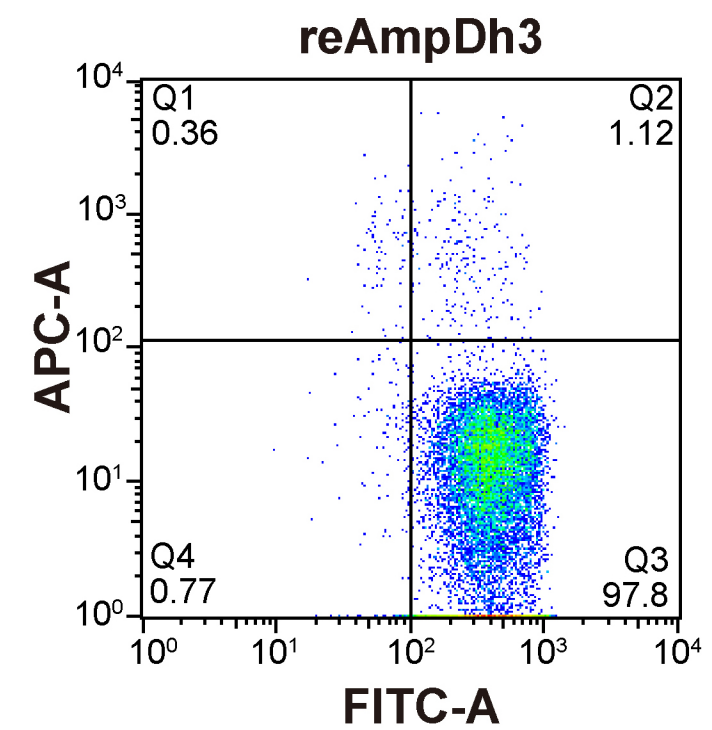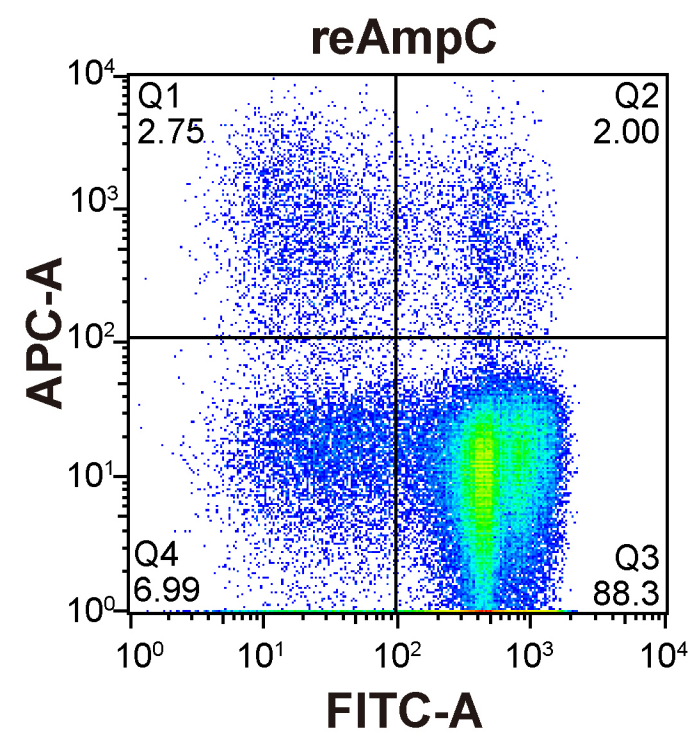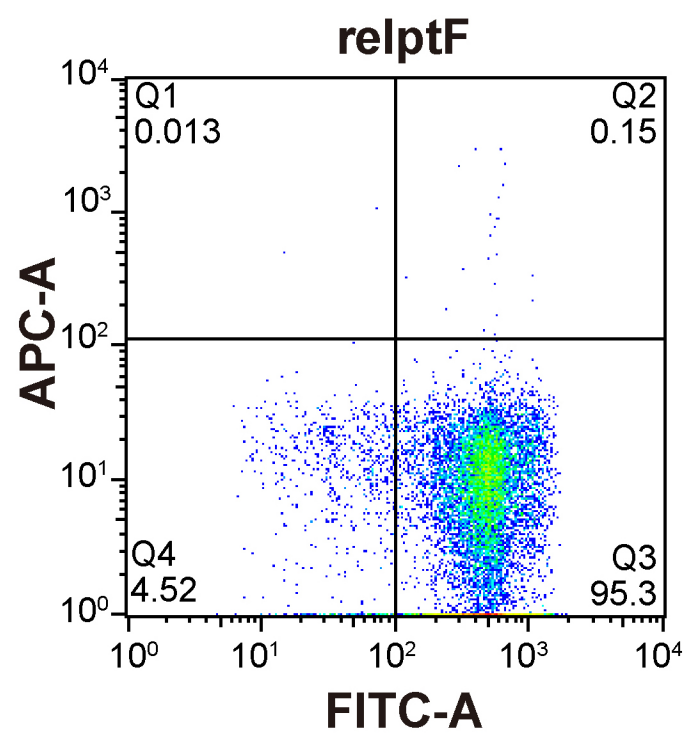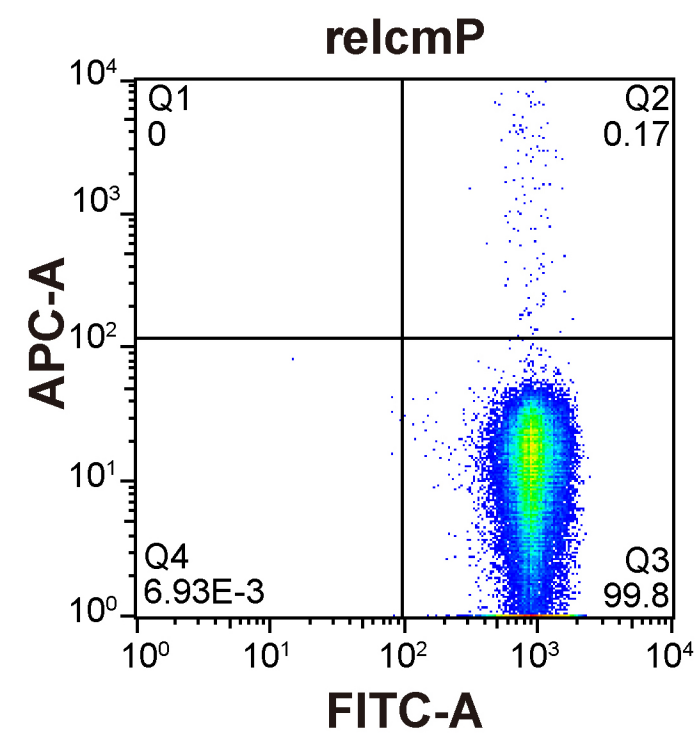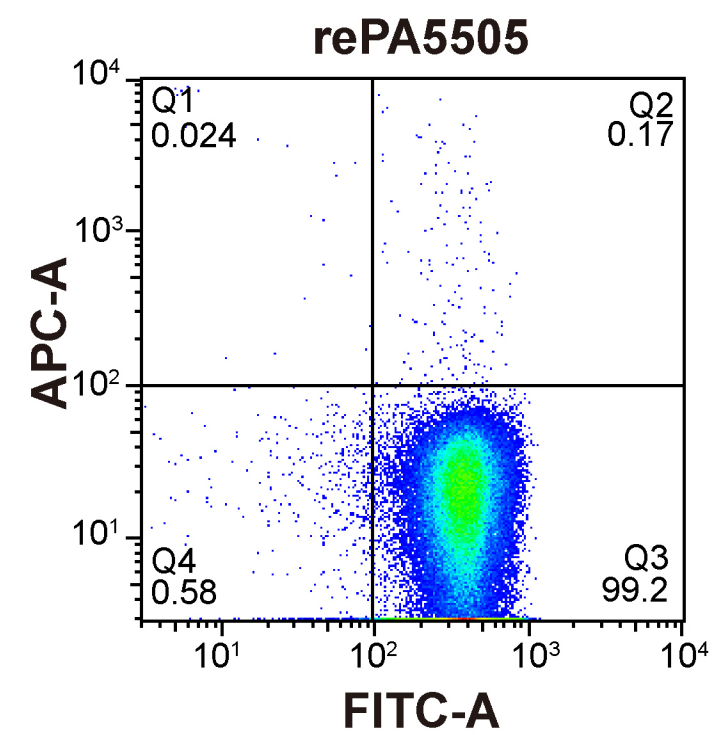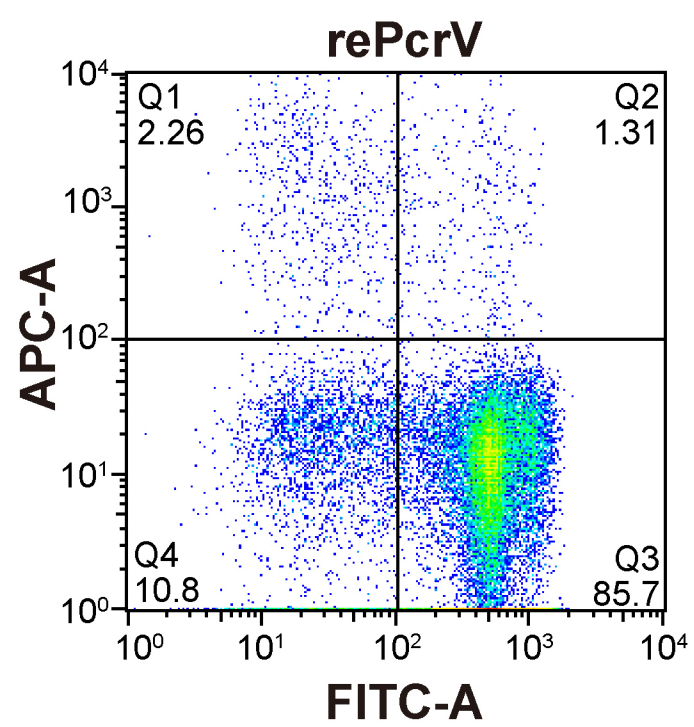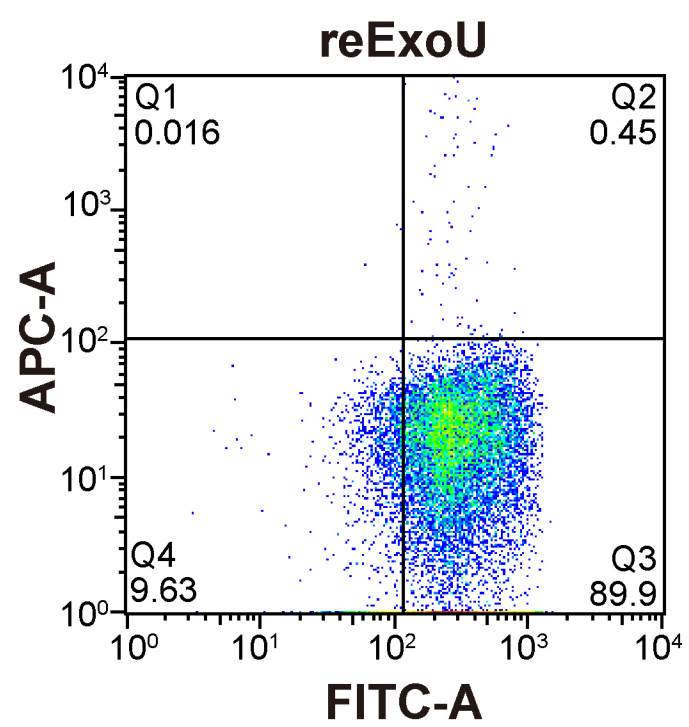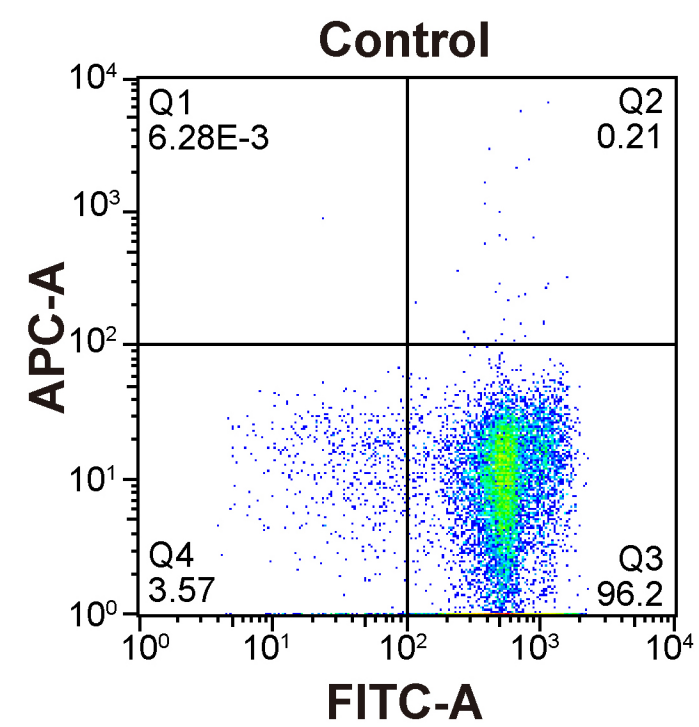

kDa

170

130

100

70

55

40

35

25

15

10

Maker

PVAC

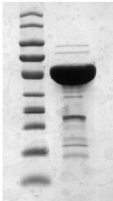

Supplement: Supplementary file 1 [file DataSheet_1.pdf]
