# Supplementary material for: Development of a Chimeric Vaccine Against Pseudomonas aeruginosa Based on the Th17-Stimulating Epitopes of PcrV and AmpC
Source: Front Immunol. 2021 Jan 21;11:601601. doi: 10.3389/fimmu.2020.601601 (PMC7859429; doi:10.3389/fimmu.2020.601601)
Supplement: Supplementary file 2 [file DataSheet_2.pdf]

Table S1 Information on the 37 candidate antigens.

No.	Ref protein	Gene description	Locus tag	Sequence	Predicited PI	Mw(kDa)	Expression in *E.coli*
1	rePstS	phosphate ABC transporter substrate-binding protein	PA5369	41Gly-323Leu	8.92	30.5	Soluble
2	reFlgE	flagellar hook protein FlgE	PA1080	1Met-462Arg	4.48	48.3	Soluble
3	reOprL	peptidoglycan associated lipoprotein OprL	PA0973	22Cys-168Lys	5.73	15.8	Soluble
4	reAmpDh3	Protein AmpDh3	PA0807	1Met-255Ala	6.10	28.7	Soluble
5	reAmpC	beta-lactamase	PA4110	32Asp-397Arg	8.89	40.3	Soluble
6	reIptF	outer membrane porin F	PA3692	27Asn-261Gly	9.36	25.9	Soluble
7	rePA5505	TonB-dependent receptor	PA5505	22Ala-261Phe	6.78	26.03	Soluble
8	reIcmP	insulin-cleaving metalloproteinase outer membrane protein	PA4370	48Lys-445Phe	4.52	42.7	Soluble
9	rePcrV	type III secretion protein PcrV	PA1706	28Gly-294Ile	5.08	29.5	Soluble
10	reExoU	type III secretion system effector cytotoxin ExoU		100Ser-360Asn	8.34	27.4	Soluble
11	reOprF	Outer membrane porin F	PA1777	25Gln-350Lys	4.75	35.2	Precipitated
12	reOprI	Major outer membrane lipoprotein	PA2853	25Lys-83Lys	6.67	6.4	Precipitated
13	reEta	Exotoxin A	PA1148	401Ala-638Lys	4.68	25.6	Precipitated
14	rePlcB	phospholipase C	PA0026	23Trp-326Leu	5.10	34.5	No expression
15	rePilA	Type IV major pilin protein PilA	PA4525	30Tyr-146Gly	6.56	12.1	Precipitated
16	reAlgE	Alginate production protein AlgE	PA3544	35Ser-490Phe	4.64	50.9	No expression
17	reOprG	outer membrane protein	PA2760	22Asn-425Phe	5.12	44.6	Precipitated
18	rePopB	Translocator protein PopB	PA1708	1Met-390Ile	5.93	40.1	Precipitated
19	reFpvA	Ferripyoverdine receptor	PA2398	523Asp-815Phe	5.19	33.7	Precipitated
20	rePelC	biosynthesis outer membrane protein	PA3062	27Ala-173Glu	5.17	16.0	Precipitated
21	reAprF	Alkaline protease secretion protein	PA1248	27Leu-444Phe	5.85	47.4	No expression

No.	Ref protein	Gene description	Locus tag	Sequence	Predicited PI	Mw(kDa)	Expression in *E.coli*
22	rePA4974	Probable outer membrane protein	PA4974	31Asp -482Tyr	5.35	50.4	No expression
23	PA2462	Haemagg_act domain-containing protein	PA2462	40Asn -420Lys	7.35	39.0	Precipitated
24	reOprM	Outer membrane protein OprM	PA0427	65Gln-463Leu	5.35	43.4	No expression
25	reExoT	Exoenzyme T	PA0044	100Ser-457Ala	5.14	38.0	Precipitated
26	reExoS	Secreted exoenzyme S	PA3841	95Met-453Ala	5.33	38.3	Precipitated
27	reAzu	Azurin	PA4922	21Ala-148Lys	5.92	13.9	No expression
28	rePA3931	Uncharacterized protein	PA3931	22Glu -259Phe	6.43	26.0	No expression
29	reHasAp	Heme acquisition protein HasAp	PA3407	1Met-205Ala	4.07	20.9	Precipitated
30	reChiC	Chitinase	PA2300	25Gly-483Arg	5.17	50.4	Precipitated
31	reOprE	Anaerobically-induced outer membrane porin OprE	PA0291	30Ala-460Leu	6.52	46.7	Precipitated
32	reOprH	outer membrane protein H	PA1178	23Ala-200Phe	8.30	19.4	Precipitated
33	reOaT	O-antigen translocase	PA3153	1Met-411Lys	8.71	45.3	No expression
34	reFlgG	Flagellar basal-body rod protein FlgG	PA1082	1Met-261Leu	4.33	27.7	Precipitated
35	reDCP	DAO domain-containing protein	PA0534	32Asp -429Phe	7.09	44.3	Precipitated
36	reCntO	Metal-pseudopaline receptor CntO	PA4837	31Glu -708Tyr	5.83	76.0	No expression
37	rePopD	Translocator outer membrane protein PopD	PA1709	138Gly-295Val	9.01	17.1	Precipitated

Table S2 The general information on PA strains

**P.aeruginosa strain**	**Source**	**Serotypes**	**Lethal doses (CFU per mouse)**	**Sublethal doses (CFU per mouse)**
XN-1	Southwest Hospital in Chongqing, China	1	1.0×10^{7}	1.3×10^{6}
PA-464	Southwest Hospital in Chongqing, China	4	2.5×10^{7}	3.5×10^{6}
ZNJ004	No.422 Hospital of the Chinese People's Liberation Army	15	3.5×10^{6}	5.8×10^{5}
PA-451	Southwest Hospital in Chongqing, China	3	1.0×10^{7}	1.4×10^{6}

Number	Location	Sequence	Number	Location	Sequence
P1	1-18	DRLKALVDAAVQPVMKAN	P27	183-200	THLDVPEAALAQYAQGYG
P2	8-25	DAAVQPVMKANDIPGLAV	P28	190-207	AALAQYAQGYGKDDRPLR
P3	15-32	MKANDIPGLAVAISLKGE	P29	197-214	QGYGKDDRPLRVGPGPLD
P4	22-39	GLAVAISLKGEPHYFSYG	P30	204-221	RPLRVGPGPLDAEGYGVK
P5	29-46	LKGEPHYFSYGLASKEDG	P31	211-228	GPLDAEGYGVKTSAADLL
P6	36-53	FSYGLASKEDGRRVTPET	P32	218-235	YGVKTSAADLLRFVDANL
P7	43-60	KEDGRRVTPETLFEIGSV	P33	225-242	ADLLRFVDANLHPERLDR
P8	50-67	TPETLFEIGSVSKTFTAT	P34	232-249	DANLHPERLDRPWAQALD
P9	57-74	IGSVSKTFTATLAGYALT	P35	239-256	RLDRPWAQALDATHRGYY
P10	64-81	FTATLAGYALTQDKMRLD	P36	246-263	QALDATHRGYYKVGDMTQ
P11	71-88	YALTQDKMRLDDRASQHW	P37	253-270	RGYYKVGDMTQGLGWEAY
P12	78-95	MRLDDRASQHWPALQGSR	P38	260-277	DMTQGLGWEAYDWPISLK
P13	85-102	SQHWPALQGSRFDGISLL	P39	267-284	WEAYDWPISLKRLQAGNS
P14	92-109	QGSRFDGISLLDLATYTA	P40	274-291	ISLKRLQAGNSTPMALQP
P15	99-116	ISLLDLATYTAGGLPLQF	P41	281-298	AGNSTPMALQPHRIARLP
P16	106-123	TYTAGGLPLQFPDSVQKD	P42	288-305	ALQPHRIARLPAPQALEG
P17	113-130	PLQFPDSVQKDQAQIRDY	P43	295-312	ARLPAPQALEGQRLLNKT
P18	120-137	VQKDQAQIRDYYRQWQPT	P44	302-319	ALEGQRLLNKTGSTNGFG
P19	127-144	IRDYYRQWQPTYAPGSQR	P45	309-326	LNKTGSTNGFGAYVAFVP
P20	134-151	WQPTYAPGSQRLYSNPSI	P46	316-333	NGFGAYVAFVPGRDLGLV
P21	141-158	GSQRLYSNPSIGLFGYLA	P47	323-340	AFVPGRDLGLVILANRNY
P22	148-165	NPSIGLFGYLAARSLGQP	P48	330-347	LGLVILANRNYPNAERVK
P23	155-172	GYLAARSLGQPFERLMEQ	P49	337-354	NRNYPNAERVKIAYAILS
P24	162-179	LGQPFERLMEQQVFPALG	P50	344-361	ERVKIAYAILSGLEQQGK
P25	169-186	LMEQQVFPALGLEQTHLD	P51	351-368	AYAILSGLEQQGKVPLKR
P26	176-193	PALGLEQTHLDVPEAALA			

Number	Location	Sequence	Number	Location	Sequence
Pc1	1-18	GSEQEELLALLRSERIVL	Pc23	155-172	AKQGIRIDAGGIDLVDPT
Pc2	8-25	LLALLRSERIVLAHAGQP	Pc24	162-179	IDAGGIDLVDPTLYGYAV
Pc3	15-32	SERIVLAHAGQPLSEAQV	Pc25	169-186	DLVDPTLYGYAVGDPRWK
Pc4	22-39	AHAGQPLSEAQVLKALAW	Pc26	176-193	LYGYAVGDPRWKDSPEYA
Pc5	29-46	LSEAQVLKALAWLLAANP	Pc27	183-200	GDPRWKDSPEYALLSNLD
Pc6	36-53	LKALAWLLAANPSAPPGQ	Pc28	190-207	DSPEYALLSNLDTFSGKL
Pc7	43-60	LLAANPSAPPGQGLEVLR	Pc29	197-214	LLSNLDTFSGKLSIKDFL
Pc8	50-67	SAPPGQGLEVLREVLQAR	Pc30	204-221	TFSGKLSIKDFLSGSPKQ
Pc9	57-74	GLEVLREVLQARRQPGAQ	Pc31	211-228	SIKDFLSGSPKQSGELKG
Pc10	64-81	EVLQARRQPGAQWDLREF	Pc32	218-235	SGSPKQSGELKGLSDEYP
Pc11	71-88	RQPGAQWDLREFLVSAYF	Pc33	225-242	SGELKGLSDEYPFEKDNN
Pc12	78-95	WDLREFLVSAYFSLHGRL	Pc34	232-249	LSDEYPFEKDNNPVGNFA
Pc13	85-102	LVSAYFSLHGRLDEDVIG	Pc35	239-256	FEKDNNPVGNFATTVSDR
Pc14	92-109	SLHGRLDEDVIGVYKDVL	Pc36	246-263	PVGNFATTVSDRSRPLND
Pc15	99-116	DEDVIGVYKDVLQTQDGK	Pc37	253-270	TTVSDRSRPLNDKVNEKT
Pc16	106-123	VYKDVLQTQDGKRKALLD	Pc38	260-277	SRPLNDKVNEKTTLLNDT
Pc17	113-130	QTQDGKRKALLDELKALT	Pc39	267-284	KVNEKTTLLNDTSSRYNS
Pc18	120-137	RKALLDELKALTAELKVY	Pc40	274-291	TLLNDTSSRYNSAVEALN
Pc19	127-144	ELKALTAELKVYSVIQSQ	Pc41	281-298	SSRYNSAVEALNRFIQKY
Pc20	134-151	AELKVYSVIQSQINAALS	Pc42	288-305	AVEALNRFIQKYDSVLRD
Pc21	141-158	SVIQSQINAALSAKQGIR	Pc43	295-312	RFIQKYDSVLRDILSAI
Pc22	148-165	INAALSAKQGIRIDAGGI			
